# Riboflavin (VB2) inhibits hepatocellular carcinogenesis by enhancing retinol metabolism and suppressing cell proliferation in Hras12V transgenic mice

**DOI:** 10.3389/fonc.2026.1773897

**Published:** 2026-03-11

**Authors:** Jiayu Song, Ning Wang, Nan Mao, Jun Chen, Rujiao Jiang, Aiguo Wang, Huiling Li

**Affiliations:** Department of Comparative Medicine, Laboratory Animal Center, Dalian Medical University, Dalian, Liaoning, China

**Keywords:** cell proliferation, hepatocellular carcinoma (HCC), Hras12V, retinol metabolism, riboflavin (VB2), tumorigenesis

## Abstract

**Aims:**

Riboflavin (VB2) is primarily utilized as an adjuvant in cancer therapy. This study aims to investigate the preventive and therapeutic effects of VB2 alone on hepatocellular carcinoma (HCC).

**Main methods:**

The preventive and therapeutic efficacy of VB2 against HCC was evaluated using a Hras12V transgenic mouse model of HCC. Initial mechanistic insights were obtained through transcriptome sequencing combined with bioinformatic analyses, and key findings were validated via molecular biology techniques.

**Key findings:**

VB2 administration significantly suppressed hepatic tumorigenesis, as evidenced by reductions in liver tumor burden and improved histology. Bioinformatic analysis revealed that VB2-mediated tumor suppression may involve the regulation of multiple metabolic pathways, including fatty acid and amino acid metabolism. Subsequent molecular validation indicated that VB2 enhanced hepatic retinol metabolism by upregulating key metabolic enzymes. It concurrently inhibited hepatocellular proliferation through p21-mediated G1/S phase arrest and suppressed DNA replication by downregulating the Mcm helicase complex. Additionally, VB2 exhibited inhibitory activity against the progression of established tumors, although this effect was not as significant as its suppression of hepatic tumorigenesis. Safety assessments in wild-type C57BL/6 mice revealed no significant treatment-related toxicity.

**Significance:**

To our knowledge, this study is the first to demonstrate *in vivo* that VB2 alone can significantly suppress hepatic tumorigenesis by enhancing retinol metabolism and inhibiting cell proliferation pathways, highlighting its potential as a chemopreventive agent for HCC.

## Highlights

- While VB2 has been studied as an adjuvant, our work is, to our knowledge, the first to demonstrate its efficacy as a single agent in preventing HCC *in vivo*.
- Establishes a transcriptomic database for exploring VB2’s anti-HCC mechanism.
- VB2 suppresses HCC genesis by activating the retinol metabolism axis.
- VB2 inhibits HCC genesis via P21-dependent G1/S arrest and Mcm downregulation to impede proliferation.
- VB2 shows a favorable safety profile at the therapeutic dosage (170 mg/kg/day).

## 1 Introduction

Hepatocellular carcinoma (HCC) accounting for 90% of primary liver malignancies, represents a significant global health challenge as the sixth most prevalent neoplasm and the third leading contributor to cancer-related mortality worldwide (1–3). Contemporary epidemiological analyses with 906, 000 diagnosed cases recorded globally in 2020, accompanied by a discouraging relative five-year survival rate of merely 18% (4), reveal an alarming disease burden.

Currently, surgical resection is the preferred treatment for early-stage HCC patients with good liver function, achieving a 5-year overall survival rate of 58.8% (5). However, the high postoperative recurrence rate remains a major clinical challenge, with 50%-70% of patients experiencing recurrence within 5 years, and about half of these recurrences occurring within the first 2 postoperative years (6–8). These findings highlight the urgent need to develop effective recurrence prevention strategies.

Riboflavin (Vitamin B2, VB2) is a water-soluble micronutrient essential for human physiology (9, 10). As the biochemical precursor of flavin mononucleotide (FMN) and flavin adenine dinucleotide (FAD), VB2 plays a critical role in cellular metabolism such as mitochondrial electron transport, redox reactions (11–13). Additionally, the clinical significance of VB2 extends beyond its metabolic functions, with therapeutic applications documented in pediatric neuropathy and migraine prophylaxis at high dosages (14). In oncology, research on VB2 has primarily centered on its association with deficiency-related oncogenic risk, its utility as an adjuvant to enhance the efficacy or mitigate the toxicity of chemotherapeutic agents such as cisplatin (15–17), and its application as a photosensitizer in photodynamic therapy (18). Notably, the standalone anti-tumor efficacy of high-dose VB2 at pharmacologically relevant concentrations has not been systematically investigated *in vivo*, particularly in HCC models that recapitulate the complete human tumorigenesis cascade.

To address this research gap, we administered high-dose VB2 (170 mg/kg/day) with biological safety to investigate its potential anti-neoplastic effects and underlying mechanisms in HCC pathogenesis by using the Hras12V transgenic HCC model that recapitulates the complete HCC cascade from initiation to malignant progression. This study expands VB2’s potentially therapeutic repertoire beyond current adjuvant uses.

## 2 Materials and methods

### 2.1 Animals and ethical statement

All mice were housed in the Laboratory Animal Center of Dalian Medical University under specific pathogen-free (SPF) conditions with a 12-hour light/dark cycle, temperature maintained at 22 ± 2 °C, and humidity at 50 ± 10%. Animals had free access to autoclaved standard laboratory chow and filtered water *ad libitum*. Experimental protocols were approved by the Institutional Animal Care and Use Committee (IACUC) of Dalian Medical University (Ethical Approval No. L20160315) and strictly adhered to the National Institutes of Health Guide for the Care and Use of Laboratory Animals. All efforts were made to minimize animal suffering and reduce the number of animals used, in compliance with the 3R principles (Replacement, Reduction, Refinement).

### 2.2 Hras12V transgenic mice model and treatment

The Hras12V transgenic mouse, a well-characterized hepatocellular carcinoma (HCC) model with male predominance in our laboratory, harbors hepatocyte-specific expression of the Hras12V oncogene (19, 20). Tumorigenesis initiates at 3–4 months of age, with macroscopic tumors developing by 6–7 months. To evaluate VB2 efficacy in both hepatic tumor initiation and progression, we designed the following experiments. Age-, litter-, and sex-matched Hras12V transgenic male mice were randomly allocated into VB2-treated and vehicle control groups using a random number generator. VB2 (170 mg/kg/day) was administered by oral gavage for 2 months, starting at either 4 months of age (tumor initiation stage) or 6 months of age (tumor progression stage). For safety assessment, age-matched wild-type C57BL/6 male mice (4-month-old, Liaoning Changsheng Biotechnology Co., Ltd., Liaoning, China) were gavaged with the same VB2 dosage for 2 months. Body weight was monitored throughout the study. All mice were euthanized at the 2-month post-treatment for subsequent histopathological and molecular analyses. The overall experimental timeline is summarized in **Supplementary Figure 1**.

### 2.3 Tissue sampling

Mice were weighed and anesthetized, followed by retro-orbital blood collection. Cervical dislocation was used for euthanasia, after which livers were dissected and collected. Liver weight, tumor size, and tumor number were meticulously recorded. For a subset of liver samples, tumor tissue (T) and peritumoral tissue (P) were dissected, snap-frozen in liquid nitrogen, and stored for subsequent RNA and protein analyses. Remaining liver tissues were fixed in 4% formalin for pathological evaluation. The researchers performing tumor counting and histological assessments were blinded to group allocation (VB2-treated group vs. the control group). All samples were coded prior to analysis, and the codes were not revealed until after all quantitative and pathological evaluations had been completed.

### 2.4 Hematoxylin-eosin staining and immunohistochemical staining

Formalin-fixed samples were paraffin-embedded and sectioned into 5-μm slices. H&E staining was performed, and sections were examined under a microscope at 40× and 200× magnifications for pathological analysis.

For IHC, sections underwent deparaffinization, hydration, heat-induced antigen retrieval in citrate buffer (pH 6.0), and blocking of endogenous peroxidase activity with 3% hydrogen peroxide. Primary and secondary antibodies were applied sequentially, followed by 3, 3’-diaminobenzidine (DAB) staining and hematoxylin counterstaining. Sections were visualized under a microscope. Antibody details are listed in **Supplementary Table 1**.

### 2.5 Hematological analysis

Hematological parameters including white blood cell (WBC), neutrophil (NEU), lymphocyte (LYM), monocyte (MONO) counts, red blood cell (RBC) count, mean platelet volume (MPV), red cell distribution width (RDW), mean corpuscular hemoglobin (MCH), mean corpuscular volume (MCV), hemoglobin (HGB), mean corpuscular hemoglobin concentration (MCHC), and platelet count (PLT) were measured using a veterinary blood cell analyzer (BC-5000 Vet, Shenzhen Mindray Animal Medical Technology Co., Ltd., China).

### 2.6 Blood chemistry measurements

Serum levels of liver function markers [alanine aminotransferase (ALT) and aspartate aminotransferase (AST)] and renal function markers [creatinine (Crea) and urea] were measured using an automatic biochemical analyzer (BS-180, Shenzhen Mindray Animal Medical Technology Co., Ltd., China).

### 2.7 RNA sequencing procedure

Total RNA was extracted using the mirVana™ miRNA Isolation Kit (Ambion, Cat# AM1561), followed by paired-end sequencing library preparation with the TruSeq^®^ RNA Sample Preparation Kit (Illumina, USA). The constructed libraries were subjected to rigorous quality control, including: quantification via Qubit^®^ 2.0 Fluorometer (Life Technologies, USA); size distribution analysis using an Agilent 2100 Bioanalyzer (Agilent Technologies, USA) to ensure insert size integrity; and molar concentration determination for precise normalization. Libraries were standardized to 10 pM, clustered on the cBot system, and sequenced on an Illumina HiSeq X Ten platform (Illumina, USA). All steps from library preparation through sequencing were conducted within a single experimental batch to preclude batch effects.

Gene expression levels were quantified using Fragments Per Kilobase of transcript per Million mapped reads (FPKM). Differential expression analysis was performed using DESeq2, with P-values adjusted for multiple testing via the Benjamini-Hochberg method to yield q-values. Differentially expressed genes (DEGs) were defined with thresholds of log2 (Fold change) > 1 and q < 0.05: the log2 (Fold change) threshold ensures biologically meaningful expression changes (≥2-fold), while the q-value threshold controls the false discovery rate (FDR) at 5%, balancing statistical rigor and biological relevance. Base calling accuracy was assessed using the Q20 metric (≥99% base call accuracy).

Bioinformatic analyses, including heatmaps, volcano plots, Gene Ontology (GO), and Kyoto Encyclopedia of Genes and Genomes (KEGG) pathway enrichment, were conducted using OECloud tools (https://cloud.oebiotech.com/). Separately, KEGG pathway enrichment for upregulated and downregulated differentially expressed genes (DEGs) was performed in Hiplot Pro (https://hiplot.com.cn/).

### 2.8 Reverse transcription-quantitative polymerase chain reaction

Total RNA was extracted from peri-tumor tissues using RNAkey Reagent (Seven Biotech, Beijing, China). One microgram of total RNA was reverse transcribed into single-stranded cDNA using the All-in-one First Strand cDNA Synthesis Kit II (with dsDNase; Seven Biotech). Quantitative PCR (qPCR) was performed using 2×SYBR Green qPCR MasterMixII (Universal; Seven Biotech) on a StepOnePlus Real-Time PCR System (Applied Biosystems) following the manufacturer’s protocols. The relative expression levels of target genes were calculated using the 2−ΔΔCt method, normalized to the housekeeping gene GAPDH. Primer sequences for target genes are listed in **Supplementary Table 2**.

### 2.9 Western blot analysis

Peri-tumor tissue proteins were extracted using RIPA Lysis Buffer (Invent Biotech, Beijing, China), and protein concentrations were quantified with a Bio-Rad xMarkmicroplate spectrophotometer. For separation, 30 µg of each protein sample was loaded onto 10% polyacrylamide gels, followed by transfer to PVDF membranes (Millipore, USA). Membranes were blocked with 5% skim milk, then incubated with primary antibodies overnight at 4 °C on a shaker. After washing with TBST, secondary antibodies were applied, followed by additional TBST washes. Primary and secondary antibodies used are listed in **Supplementary Table 1**.

### 2.10 Statistical analysis

Statistical analyses were performed using GraphPad Prism 9, with data presented as mean ± SEM. Gaussian distribution was confirmed prior to analysis: normally distributed data were analyzed by Student’s t-test, while non-normally distributed data were evaluated using the Mann-Whitney U test. A P value < 0.05 was considered statistically significant. Sample sizes ranged from n=3 to n=8 across experiments, reflecting differing technical requirements: molecular assays (e.g., Western blot, RT-qPCR) used n=3–5 replicates for reliable detection, while phenotypic assessments (e.g., tumor counting, histology) used n=5–8 to ensure statistical power. Sample sizes were based on established literature protocols and pilot data; formal power calculations were not performed as this was a feasibility and mechanism-discovery study.

## 3 Results

### 3.1 VB2 inhibits hepatic tumorigenesis

**Figure 1:**
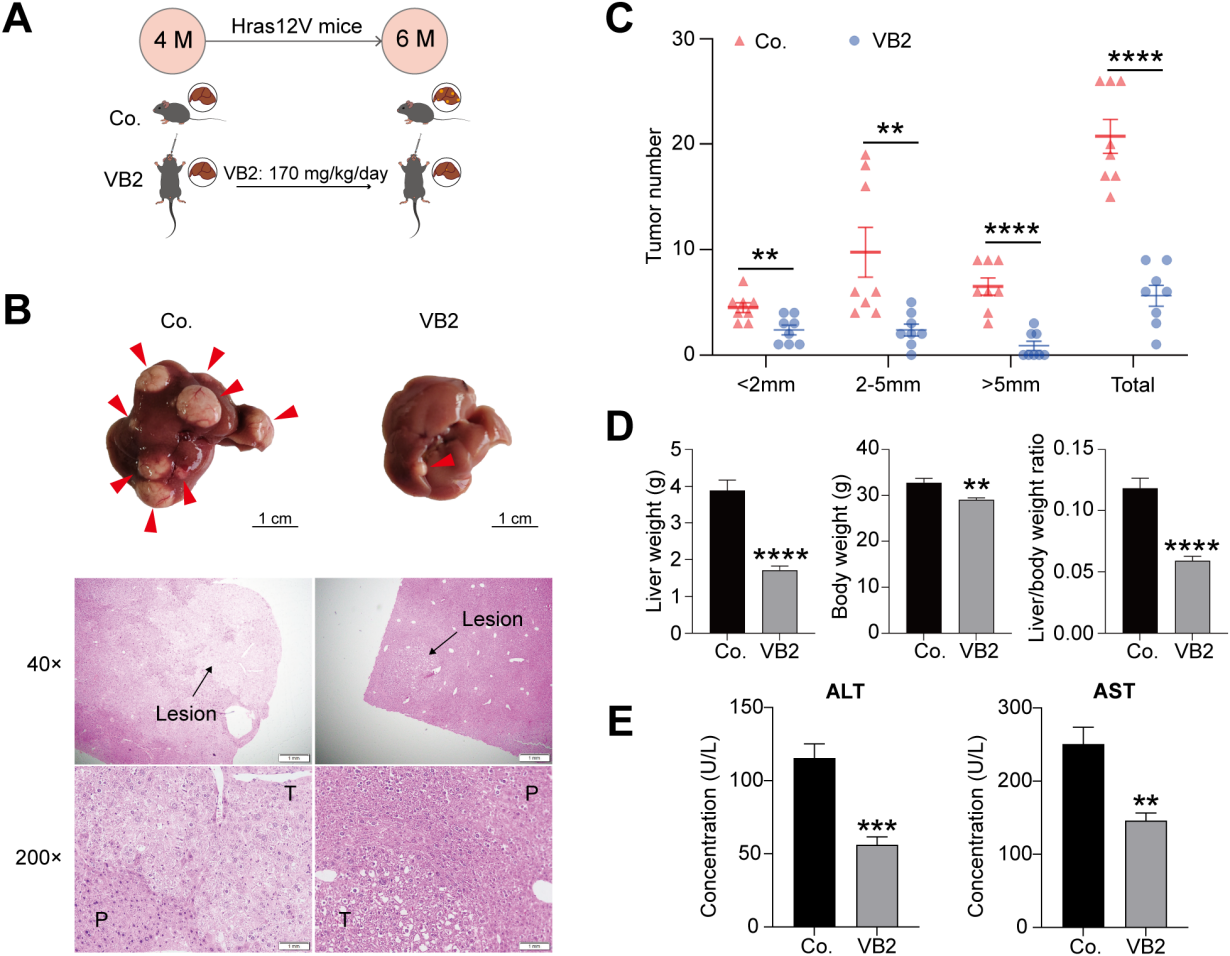
VB2 significantly suppresses hepatic tumorigenesis. **(A)** Illustration of the VB2 dosing regimen in Hras12V mice. **(B)** Representative liver stereogram and H&E stained histopathological Images (40х and 200х magnifications). The red arrow points to the hepatic tumor. **(C)** Tumors quantification by diameter. **(D)** Comparison of liver weight, body weight, and liver/body weight ratio between control (Co.) and VB2–treated groups. **(E)** Serum levels ALT and AST levels in Co. and VB2 groups. M, month; Co., control group; VB2, VB2-treated group; P, peri-tumor; T, tumor; AST, aspartate aminotransferase; ALT, alanine aminotransferase. Data presented as mean ± SEM. **p< 0.01; ***p< 0.001; ****p< 0.0001; n=5-8.

To explore the impact of VB2 on hepatic tumorigenesis, 4-month-old Hras12V mice were administered 170 mg/kg/day of VB2. After 2 months, liver pathology was examined (**Figure 1A**). Anatomical findings indicated fewer hepatic tumors in the VB2-treated group compared to the control (**Figure 1B**). Statistically, the number of tumors was markedly reduced in the VB2 group across all size categories(<2mm, 2~5mm, >5mm and total) relative to the control (**Figure 1C**). Similarly, the liver weight, body weight, and liver-to-body weight ratio were significantly lower in the VB2 group than in the controls (**Figure 1D**). H&E staining corroborated these results (**Figure 1B**). Correspondingly, the serum levels of ALT and AST were significantly decreased in the VB2 group (**Figure 1E**). Collectively, these data suggest that VB2 has an a robust inhibitory effect on hepatic tumorigenesis.

### 3.2 VB2 suppresses hepatic tumor progression

To investigate the effect of VB2 on hepatic tumor growth, 6-month-old Hras12V mice were given VB2 at a dose of 170 mg/kg/day. After 2 months, liver pathology was analyzed (**Supplementary Figure 2A**). Macroscopic observations revealed that the VB2-treated mice had smaller hepatic tumors compared to the control group (**Supplementary Figure 2B**). Consistently, both the liver weight and the liver-to-body weight ratio were notably decreased in the VB2-treated mice relative to the controls (**Supplementary Figure 2D**). Quantitatively, the number of tumors larger than 5 mm in diameter was significantly reduced in the VB2-treated group compared to the control (**Supplementary Figure 2E**). In parallel, the serum levels of ALT and AST were significantly lower in the VB2-treated group (**Supplementary Figure 2C**). Collectively, these results indicate that VB2 effectively inhibits hepatic tumor development.

### 3.3 Sequencing data general characteristics

VB2 shows inhibitory effects on both hepatic tumorigenesis and development, with its role in tumorigenesis being more prominent. Hence, the subsequent study focuses on exploring the underlying mechanisms of VB2’s inhibitory effect on hepatic tumorigenesis through transcriptome sequencing.

**Figure 2:**
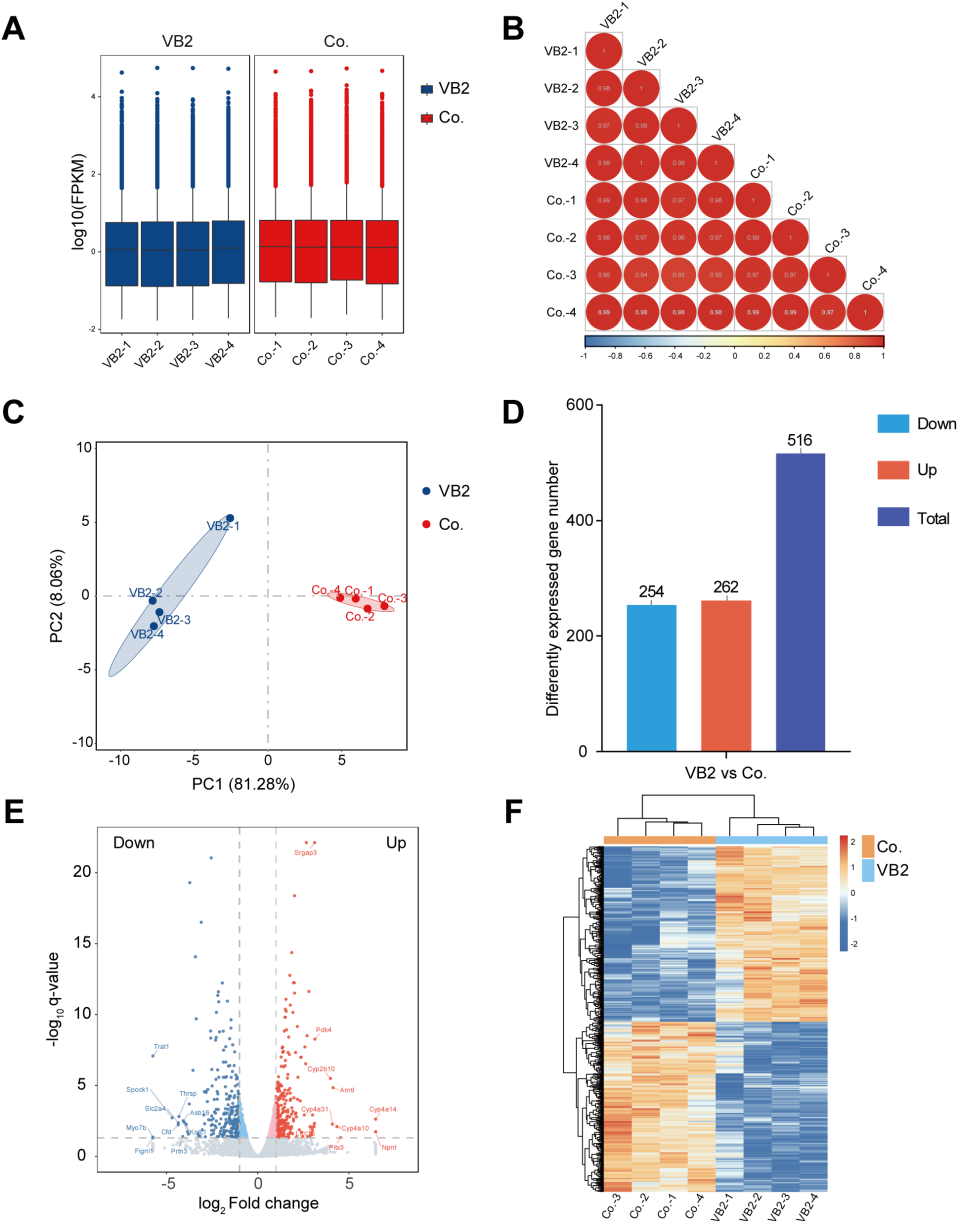
Quality of sequencing data and gene expression profile induced by VB2. **(A)** Boxplot of FPKM values. **(B)** Correlation analysis of sequencing samples. **(C)** Principal component analysis of sequencing samples. **(D)** Counts of up-regulated, down-regulated, and total differentially expressed genes (DEGs) in the VB2-treated group compared to the control group. **(E)** Volcano plot of significant DEGs between the VB2-treated and control groups. The x-axis shows log2 (Fold change), and the y-axis represents –log10(q value) for each significant DEG. **(F)** Heatmap of significant DEGs. FPKM, fragments per kilobase of exon model per million mapped fragments; DEGs, differentially expressed genes; Co., control group; VB2, VB2-treated group.

After filtering out low-quality reads, 366, 012, 472 clean reads were retained for further analysis. The percentage of bases with a Phred value above 30 (Q30) in each sample was at least 96.36%, and the GC content ranged from 47.62% to 49.16% (**Supplementary Table 3**). When comparing each sample’s reads to the reference genome, the alignment rate was 91.94% - 92.35% (**Supplementary Table 4**). The distribution of FPKM was examined, revealing generally consistent gene expression levels across samples (**Figure 2A**). Moreover, sample correlation tests and principal component analysis demonstrated significant between-group differences and high within-group consistency (**Figures 2B, C**). These results indicate that the sequencing data of both sample groups are of high quality and suitable for subsequent transcriptomic analysis.

### 3.4 VB2 triggered a differential gene expression profile

A comparison of the gene expression profiles between the VB2-treated and control groups was carried out. Using log2 (Fold change) >1 and q < 0.05 as criteria, 516 differentially expressed genes were detected, with 254 being down-regulated and 262 up-regulated (**Figures 2D, E**; **Supplementary Table 5**). Subsequently, a heat map analysis was executed to contrast these differentially expressed gene sets. In the heat map, a redder shade signified a higher expression level, and a bluer shade denoted a lower one (**Figure 2F**). Collectively, these results suggest that VB2 induces significant changes in gene expression profiles.

### 3.5 Enriched pathways in VB2-mediated inhibition of hepatic tumorigenesis

**Figure 3:**
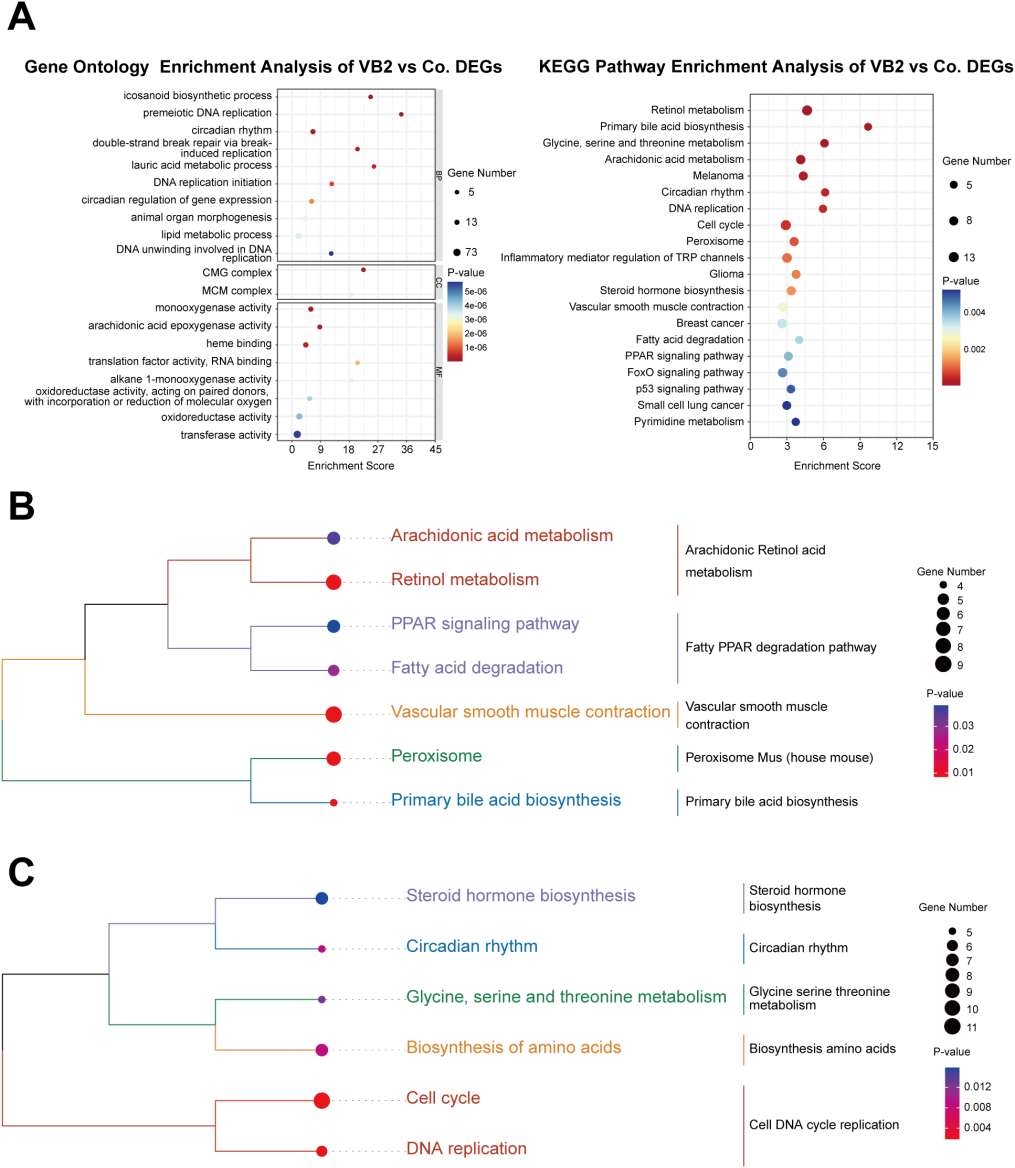
Pathways enriched in VB2-mediated inhibition of hepatic tumorigenesis. **(A)** GO and KEGG Pathway Enrichment Bubble Plots for DEGs, depicting the comparison between the VB2 and Co. groups. Each bubble represents a unique enrichment pathway, with its size proportional to the number of enriched DEGs. A redder bubble indicates a more significant role of VB2 in that pathway. **(B)** KEGG Pathway Enrichment Cluster Dendrogram for Up-Regulated DEGs, illustrating the relationships among the enriched pathways of up-regulated genes in the VB2 compared to the Co. **(C)** KEGG Pathway Enrichment Cluster Dendrogram for Down-Regulated DEGs, demonstrating the clustering of enriched pathways for down-regulated genes in the VB2 relative to the Co. DEGs, differentially expressed genes; GO, Gene Ontology; MF, Molecular Function; CC, Cellular Component; BP, Biological Process; KEGG, Kyoto Encyclopedia of Genes and Genomes; Co., control group; VB2, VB2-treated group.

To uncover the crucial pathways through which VB2 suppresses hepatic tumorigenesis, a functional enrichment analysis was performed on DEGs to clarify their biological functions (**Supplementary Tables 6**, **7**). The top 20 enriched Gene Ontology (GO) and Kyoto Encyclopedia of Genes and Genomes (KEGG) pathways are presented graphically in a bubble plot (**Figure 3A**; **Supplementary Table 8**, ).

In the GO enrichment, the emphasis is mainly on biological processes, encompassing key pathways like DNA replication, circadian rhythm, and lipid metabolism. In terms of molecular function, it chiefly involves oxidoreductase activity. The KEGG enrichment pathways mainly comprise amino acid metabolism, nucleotide metabolism, lipid metabolism, cell growth and death, all of which are closely associated with hepatic tumorigenesis.

Furthermore, to gain a deeper understanding of the pathways involved in VB2’s inhibition of hepatic tumorigenesis, cluster dendrogram analysis was carried out on the KEGG enrichment pathways of both up-regulated and down-regulated DEGs (**Figures 3B, C**). Among the up-regulated enrichment pathways, notable enrichments were detected in pathways like retinol metabolism, fatty acid degradation, and peroxisomal pathways. In contrast, within the down-regulated enrichment pathways, significant suppressions were observed in crucial metabolic and cellular regulatory pathways, such as amino acid biosynthesis and metabolism, as well as DNA replication and cell cycle control. Collectively, the combined effect of these up-regulated and down-regulated enriched pathways contributes to the suppression of hepatic tumorigenesis.

### 3.6 VB2 potentiates retinol metabolic pathways

**Figure 4:**
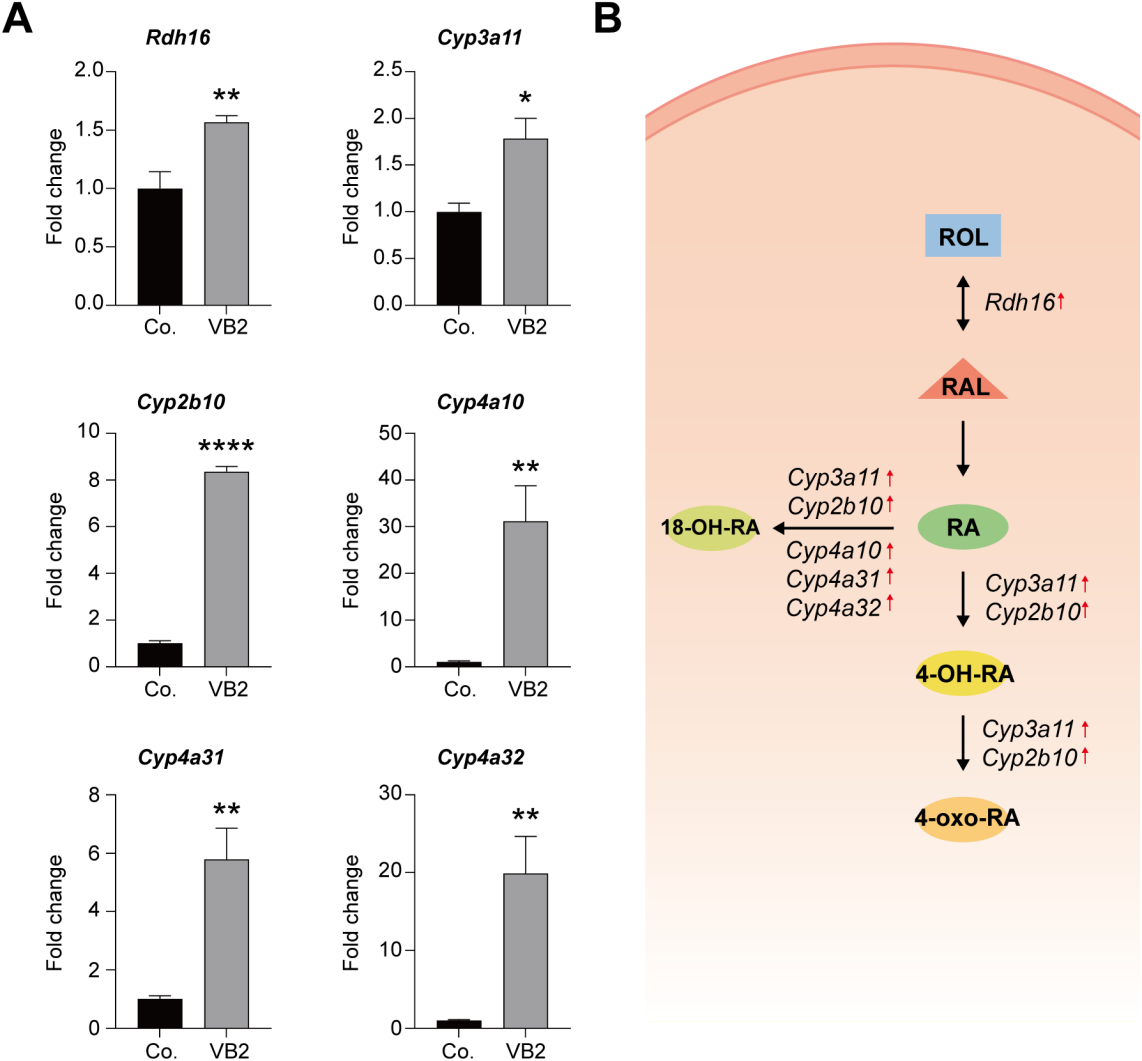
VB2 potentiates retinol metabolic pathways. **(A)** RT-qPCR was used for quantitative analysis of mRNA expression levels of key enzymes (*Rdh16*, *Cyp3a11*, *Cyp2b10*, *Cyp4a10*, *Cyp4a31*, and *Cyp4a32*) in the retinol metabolic pathway. **(B)** Summary of the enhanced retinol metabolic pathway. Co., the control group; VB2, the VB2-treated group. Data are presented as mean ± SEM. *p < 0.05; **p < 0.01; ****p < 0.0001; n = 5.

Transcriptomic analysis suggests that VB2 significantly upregulates the retinol metabolic pathway. To verify this, RT-qPCR was performed to assess the expression levels of key enzymes *Rdh16*, *Cyp3a11*, *Cyp2b10*, *Cyp4a10*, *Cyp4a31*, and *Cyp4a32* in the retinol metabolic pathway. Results showed that the expression levels of these genes were significantly higher than those in the control group (**Table 1**; **Figures 4A, B**), indicating that VB2 may exert its anti-tumor effect by enhancing retinol metabolism. This dual induction of RA synthesis (*Rdh16*) and catabolism (*CYPs*) likely represents an activated metabolic state, where bioactive RA metabolites (e.g., 4-OH-RA, 18-OH-RA, 4-oxo-RA) retain anti-neoplastic potential and contribute to tumor suppression.

**Table 1:** DEGs associated with pathways confirmed in **Figures 4** and **5**.

Gene symbol	Full name	Fold change	P-value	UP/Down
Retinol metabolism				
Rdh16	Retinol dehydrogenase 16	2.07E+00	5.20E-04	Up
Cyp3a11	Cytochrome P450, family 3, subfamily a, polypeptide 11	2.25E+00	4.64E-04	Up
Cyp2b10	Cytochrome P450, family 2, subfamily b, polypeptide 10	1.58E+01	1.91E-08	Up
Cyp4a10	cytochrome P450, family 4, subfamily a, polypeptide 10	2.03E+01	2.79E-04	Up
Cyp4a31	cytochrome P450, family 4, subfamily a, polypeptide 31	1.71E+01	1.69E-04	Up
Cyp4a32	cytochrome P450, family 4, subfamily a, polypeptide 32	3.81E+00	1.04E-05	Up
Cell cycle				
Cdkn1a	Cyclin dependent kinase inhibitor 1A	5.20E+00	4.14E-10	Up
DNA replication				
Mcm2	Minichromosome maintenance complex component 2	3.82E-01	2.44E-03	Down
Mcm3	Minichromosome maintenance complex component 3	4.64E-01	8.26E-04	Down
Mcm4	Minichromosome maintenance complex component 4	4.41E-01	2.20E-04	Down
Mcm5	Minichromosome maintenance complex component 5	1.40E-01	2.60E-07	Down
Mcm6	Minichromosome maintenance complex component 6	3.50E-01	3.38E-03	Down

### 3.7 VB2 inhibits cell proliferation

**Figure 5:**
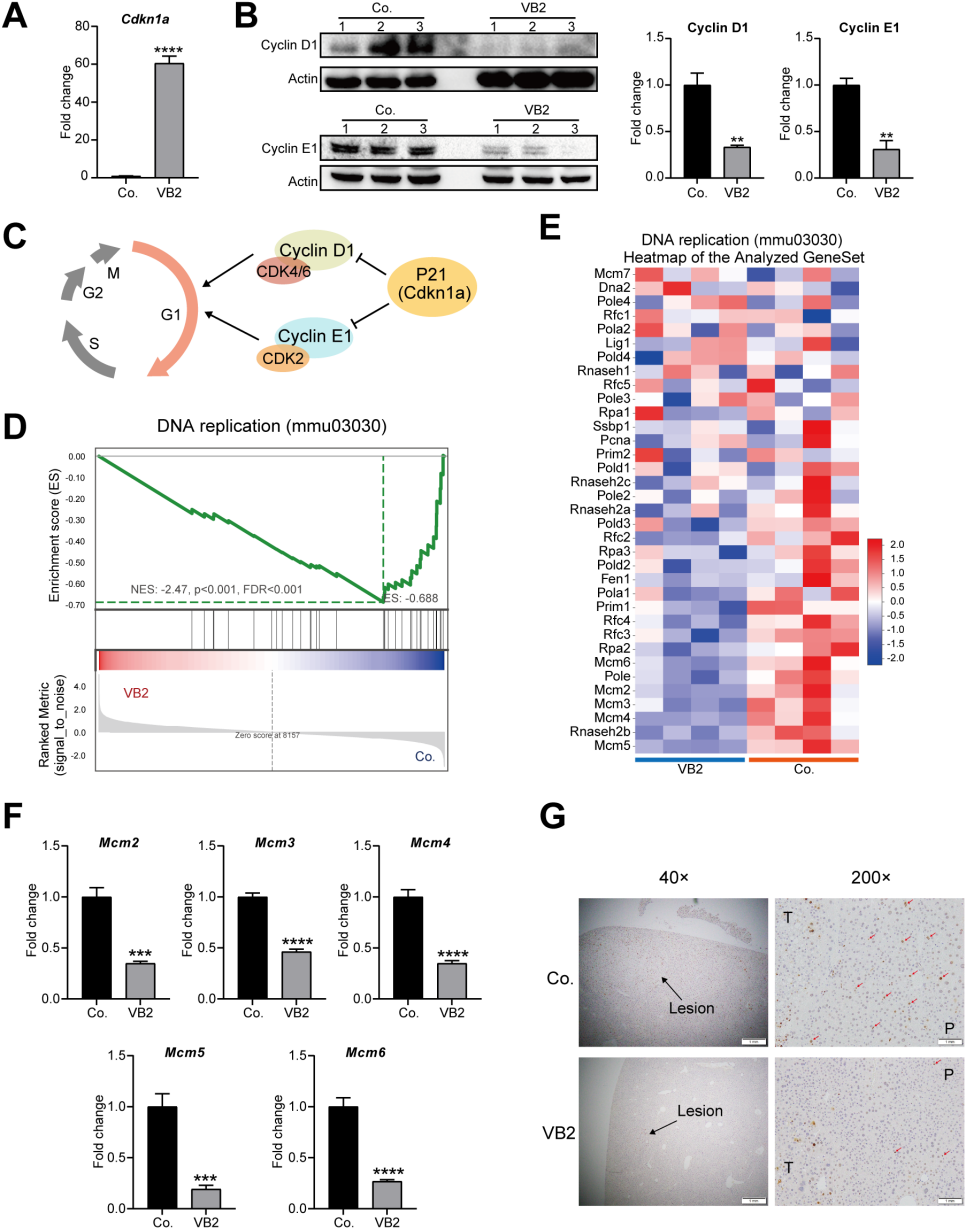
VB2 inhibits cell proliferation. **(A)** RT-qPCR was used for the quantitative analysis of *Cdkn1a* mRNA level. **(B)** Western Blot was employed to detect the protein levels of Cyclin D1 and Cyclin E1 (top panel), and relative densitometric analysis was carried out (bottom panel). **(C)** A summary of the inhibited cell cycle pathway. **(D)** Gene Set Enrichment Analysis (GSEA) of the DNA replication pathway, based on transcriptomic data. **(E)** Heatmap analysis of the gene set for the DNA replication pathway. **(F)** RT-qPCR quantification of the mRNA expression levels of the Mcm family in the DNA replication pathway. **(G)** Representative immunohistochemical analysis of Ki-67 expression at × 40 and × 200. The strong expression of Ki-67, represented by red arrowheads. Co., control group; VB2, VB2-treated group. Data are presented as mean ± SEM. **p < 0.01; ***p < 0.001; ****p < 0.0001; n = 3-5.

Based on transcriptome analysis, the cell cycle signaling pathway was significantly inhibited under VB2 treatment. RT-qPCR results verified the significantly up-regulated mRNA level of *Cdkn1a* (**Table 1**; **Figures 5A, B**). Consistently, Western blot analysis showed that Cdkn1a-targeted downstream proteins Cyclin D1 and CyclinE1 were significantly down-regulated (**Figure 5C**). Gene Set Enrichment Analysis (GSEA) clearly suggested a significant enrichment of the DNA replication pathway (**Figure 5D**). Correspondingly, heatmap analysis of gene sets in this pathway showed that the minichromosome maintenance (Mcm) family, a key part of the DNA replication pathway, was significantly downregulated in VB2 group (**Figure 5E**). RT-qPCR analysis further confirmed the significant downregulation of mRNA levels of Mcm family members *Mcm2*, *Mcm3*, *Mcm4*, *Mcm5*, and *Mcm6* (**Table 1**; **Figure 5F**). Consistently, less Ki-67 marked cells were observed in the VB2 group (**Table 1**; **Figure 5G**). These results suggest that VB2 inhibits cell proliferation by inducing G1/S phase arrest and suppressing DNA replication, supported by the coordinated upregulation of Cdkn1a, downregulation of Cyclin D1/E1 and Mcm family members, and reduced Ki-67 positivity.

### 3.8 Biosafety of VB2

**Figure 6:**
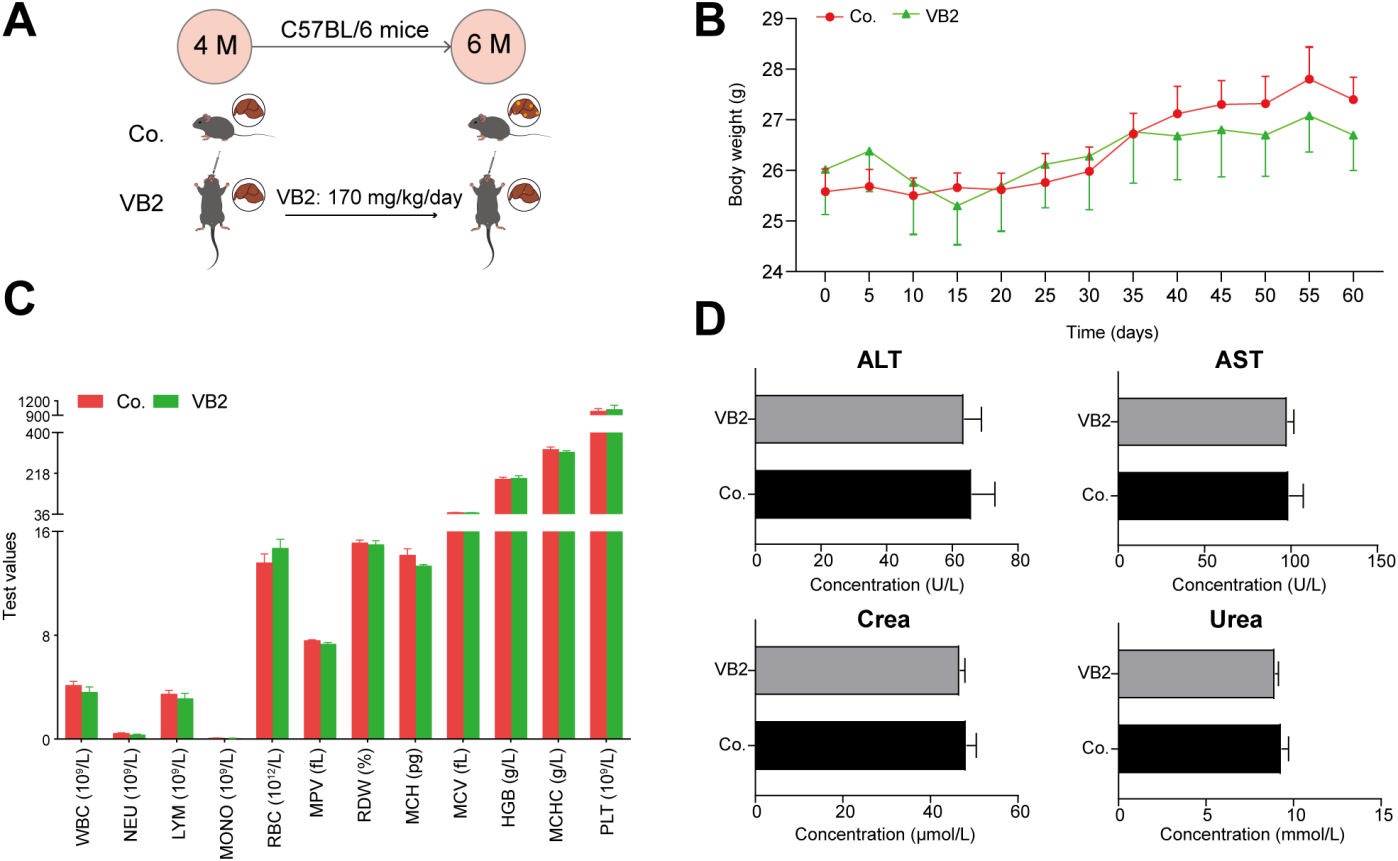
Safety of VB2 in C57BL/6 mice. **(A)** Schematic of the VB2 dosing protocol for C57BL/6 mice. **(B)** Body weight changes. **(C)** Levels of hematology-related indicators. **(D)** Serum levels of ALT, AST, Crea, and Urea. M, month; Co., control group; VB2, VB2-treated group; WBC, white blood cell; NEU, neutrophil counts; LYM, lymphocyte counts; MONO, monocyte counts; RBC, red blood cell count; MPV, mean platelet volume; RDW (%), percentage of red cell distribution width; MCH, mean corpuscular hemoglobin; MCV, mean corpuscular volume; HGB, hemoglobin; MCHC, mean corpuscular hemoglobin concentration; PLT, platelet counts; AST, aspartate aminotransferase; ALT, alanine aminotransferase. Crea, creatinine. Data are presented as mean ± SEM. n = 5.

To assess the biosafety of the VB2 dose, 4-month-old C57/BL/6 mice were administered VB2 (170 mg/kg/day) for two months (**Figure 6A**). Throughout the treatment, no significant difference in body weight was observed between the control and VB2-treated mice (**Figure 6B**). Moreover, hematology-related indicators such as WBC, NEU, LYM, MONO, RBC, MPV, RDW, MCH, MCV, HGB, MCHC, PLT, and liver- and kidney-function indicators including ALT, AST, Crea, and Urea showed no significant differences between the two groups (**Figures 6C, D**). These data suggest the biosafety of VB2 at a dose of 170 mg/kg/day for mice.

## 4 Discussion

Riboflavin (VB2), a water-soluble micronutrient essential for redox homeostasis and energy metabolism, has garnered significant attention due to its dual roles in human health: deficiency-induced pathologies and potential therapeutic applications in cancer biology. Studies have established that VB2 deficiency is associated with increased risks of esophageal, HCC and so on (21, 22). Conversely, under physiological sufficiency, VB2 predominantly functions as an adjuvant in anticancer strategies. For instance, it works in concert with vitamin C to make breast cancer cells more sensitive to vitamin C-induced cell death (23); while being used in combination with cisplatin, it reduces the hepatorenal toxicity of cisplatin by inhibiting ROS production (24). Notably, these studies underscore VB2’s auxiliary role rather than a primary therapeutic agent, with no prior evidence supporting high-dose VB2 monotherapy for solid tumors.

Pharmacological applications of supraphysiological VB2 doses have been confined to rare neurological disorders, including Brown-Vialetto-Van Laere syndrome (BVVL) and juvenile ALS-like phenotypes (25, 26), in which VB2 transporters are genetically impaired, and the prevention of migraines among others (27, 28). This study pioneers the exploration of high-dose VB2 as a standalone prophylactic and therapeutic intervention for HCC, bridging a critical gap in oncology research. Utilizing the Hras12V transgenic mouse model, a robust system recapitulating the multistep pathogenesis of HCC from dysplastic nodules to invasive carcinoma, we demonstrated that sustained VB2 administration significantly delayed tumor initiation and suppressed progression.

As a water-soluble micronutrient with limited systemic retention, VB2 undergoes rapid renal elimination, thereby establishing its characteristic low toxicity profile in oral administration. This pharmacokinetic property substantiates the prevailing consensus that VB2 exhibits negligible toxicological risks, even when consumed in quantities exceeding physiological requirements. Notably, excessive intake has been demonstrated to neither compromise dietary intake nor induce body weight alterations in mammalian models (29). Current pharmacological surveillance further corroborates this safety paradigm, with no documented cases of VB2 toxicity across clinical and preclinical studies despite supra-nutritional dosing regimens. Limited reports of transient gastrointestinal manifestations (e.g., diarrhea, vomiting) have been mechanistically attributed to saturation of gastrointestinal transport capacity rather than inherent compound toxicity (30). The upper limit of human intake of VB2 has been explored, and no adverse effects have been observed in rats fed 900 mg/kg/day of VB2 (31). Building upon this established safety framework, our experimental protocol administered 170 mg/kg/day VB2 to Hras12V transgenic mice – a dosage classified as pharmacologically elevated yet remaining below thresholds associated with transport saturation phenomena. Comprehensive toxicological monitoring revealed that at this dose, the mice were able to maintain normal growth parameters, the body weight trajectory was parallel to that of the control cohort, the relevant hematological indicators were within the normal range, and no hepatorenal toxicity was developed (**Figure 6**).

Based on body surface area normalization (32), the human-equivalent dose (HED) of the intervention is calculated to be approximately 12 mg/kg/day (corresponding to a daily dose of about 720 mg for a 60 kg adult). This pharmacological dosage is substantially higher than the recommended dietary allowance for adults (1.3–1.6 mg/day) (33) but falls within the lower range of high-dose VB2 therapy reported clinically (7–60 mg/kg/day) (34) for conditions such as migraine prophylaxis and VB2 transporter deficiency. Therefore, the proposed dosage is clinically feasible. Given its excellent safety profile, affordability, and accessibility, VB2 may warrant consideration as a potential chemopreventive agent. One clinically viable approach could be its integration into postoperative management, for instance, as a daily supplement used alongside routine surveillance, to potentially reduce HCC recurrence risk. VB2 could also be combined with antiviral therapy or metabolic interventions in high-risk populations (e.g., patients with cirrhosis) to enhance prevention efficacy.

Notably, previous studies have shown that VB2 can reduce the hepatorenal toxicity of cisplatin (24), supporting its potential utility in combination regimens. Future studies should systematically investigate the synergy between VB2 and standard HCC therapies (e.g., sorafenib, anti-PD-1 immunotherapy) to explore whether combinatorial strategies can enhance therapeutic efficacy for established tumors.

Mounting evidence has demonstrated that retinol metabolism serves as a modulator that exhibits a negative correlation with the progression of HCC. This has positioned retinol metabolism as a highly promising therapeutic target for the management of HCC (35–37). Intriguingly, our study has uncovered a novel and previously unreported mechanism by which VB2 enhances retinol metabolism pathways. Specifically, VB2 achieves this by upregulating key enzymatic regulators, namely retinal dehydrogenase 16 (Rdh16) and cytochrome P450s (CYP), as illustrated in **Figure 4**. The elevation of Rdh16 induced by VB2 is particularly noteworthy. Rdh16 is a crucial enzyme in the biosynthesis of retinoic acid (RA) (38), and this increase aligns well with the well-established tumor-suppressive properties of RA in the context of hepatic tumorigenesis (39–42). Significantly, we observed that VB2 simultaneously mediates the induction of Cyp2b, Cyp3a, and Cyp4a. This finding appears paradoxical at first glance, considering the documented role of these CYPs in the catabolism of RA (43, 44). However, it is important to note that therapeutic retinoids have been reported to paradoxically upregulate the expression of Cyp3a/4a during cancer treatment (45, 46).

We propose that this dual induction represents an activated metabolic state rather than a simple counteraction. While CYP upregulation may serve as an adaptive feedback response, it is also possible that the resulting bioactive RA metabolites, such as 4-OH-RA, 18-OH-RA, and 4-oxo-RA, which retain anti-neoplastic potential, contribute to the overall tumor-suppressive effect, as evidenced by previous research studies (47). To our knowledge, this current investigation represents the first experimental evidence suggesting that VB2 exerts preventive effects against hepatic tumorigenesis by potentiating retinol metabolism. The dual-phase metabolic modulation mechanism, which involves enhancing RA synthesis through the activation of Rdh16 and generating bioactive RA derivatives via CYP-mediated metabolism, establishes a novel and innovative paradigm for the prevention of HCC. This paradigm not only deepens our understanding of the complex interplay between VB2, retinol metabolism, and hepatic tumorigenesis but also offers new insights and potential strategies for the development of preventive and therapeutic interventions for HCC. While our transcriptomic and molecular data suggest VB2 enhances retinol metabolism, this evidence remains indirect. Direct profiling of retinoid metabolites (e.g., all-trans RA, 4-OH-RA, 4-oxo-RA) in future metabolomic studies is therefore required to validate the activation of this pathway.

VB2 exerts anti-tumor effects by regulating multiple metabolism-related signaling pathways, particularly through promoting fatty acid degradation and inhibiting amino acid synthesis, which collectively contribute to the suppression of hepatic tumorigenesis. First, VB2 significantly enhances fatty acid degradation, a process closely linked to HCC suppression. Preclinical studies have shown that increased fatty acid degradation is negatively correlated with HCC progression (48), and *in vitro* experiments have demonstrated that activating this pathway can potentiate anti-tumor efficacy (49). Consistently, our data show that VB2 treatment promotes fatty acid catabolism (**Figure 3A**; **Supplementary Table 9**), suggesting that this pathway is a key mediator of VB2-induced tumor inhibition. Second, VB2 suppresses the synthesis of amino acids, including glycine and serine, which are critical for tumor cell proliferation. Upregulated amino acid biosynthesis has been identified as a driver of HCC progression (50), and inhibiting serine/glycine metabolism has been shown to impair tumor growth and survival (51, 52). Notably, VB2 treatment leads to downregulation of glycine and serine synthesis (**Figure 3A**; **Supplementary Table 9**), indicating that VB2 restricts tumor development by depleting these essential metabolites. Together, these findings reveal a dual regulatory mechanism of VB2 in hepatic tumorigenesis: promoting catabolic pathways (fatty acid degradation) while suppressing anabolic pathways (amino acid synthesis). This coordinated modulation of metabolic networks likely deprives tumor cells of energy and building blocks, thereby inhibiting their proliferation and survival.

The cell cycle is a fundamental regulator of cell proliferation, making it a pivotal target for cancer therapy (53–55). Current studies on VB2 deficiency have uncovered a paradox in cell cycle control: while *in vitro* models show that VB2 deficiency induces protein and DNA damage leading to G1 phase arrest (56), both *in vitro* and *in vivo* evidence paradoxically demonstrates that VB2 deficiency promotes aberrant G2/M phase accumulation, thereby accelerating cell proliferation and tumorigenesis (57). Notably, to our knowledge, our study for the first time reveals that VB2 supplementation inhibits cell proliferation *in vivo*, as evidenced by a significant reduction in the Ki67-positive cell rate (**Figure 5**). Notably, Ki-67 staining in this study was a qualitative and semi-quantitative assessment to confirm the anti-proliferative trend, with more rigorous quantitative scoring to be applied in future research. This antiproliferative effect is mediated by G1/S phase arrest, achieved through upregulation of the cell cycle inhibitor P21 and concurrent downregulation of Cyclin D1/E1 (**Figure 5**). Additionally, the downregulation of Mcm family proteins-key initiators of DNA replication-likely contributes to the suppression of S phase progression (**Figure 5**). These findings resolve the previously observed paradox by demonstrating that VB2 exerts its antiproliferative effect through dual regulation of the cell cycle: blocking G1/S transition and inhibiting DNA replication. This mechanism provides a novel rationale for targeting VB2 metabolism in cancer therapy, highlighting its potential as a therapeutic strategy to disrupt abnormal cell cycle progression in tumors. Notably, while these findings collectively delineate a coherent pathway from p21 upregulation to cell cycle arrest, we acknowledge the inherent challenge in dissecting direct drug effects from secondary consequences within the complex tumor microenvironment. The observed p21 induction may be partly attributable to reduced tumor burden. Future studies employing *in vitro* models (e.g., HCC cell lines treated with VB2) will be essential to conclusively establish VB2 as a direct inducer of p21 and to validate the causal sequence of this mechanism.

This study showed VB2 significantly inhibits hepatic tumor initiation, but its effect on tumor progression is limited. However, the mechanisms underlying this differential effect remain unclear and warrant further investigation. Furthermore, the present work examined only a single dose of VB2, without performing a systematic dose-response analysis to delineate its efficacy-safety profile. Future studies should therefore include dose-ranging experiments to optimize dosing strategies and define the therapeutic window. Additionally, our conclusions are derived from a specific sex context, and future studies should formally evaluate potential sex-dependent effects of VB2. To enhance therapeutic efficacy, future research should explore combinatorial strategies of VB2 with other agents. Additionally, while transcriptome sequencing identified potential mechanisms, the molecular basis remains underexplored. In-depth studies integrating functional assays are needed to clarify the underlying pathways and optimize VB2-based therapeutic approaches.

The choice to perform RNA-seq on peri-tumoral tissue rather than tumor tissue was intentional, as our primary objective was to identify the early preventive mechanisms of VB2 against hepatocarcinogenesis. Peri-tumoral tissue, while histologically normal or pre-neoplastic, resides within the pro-tumorigenic field and represents the cellular environment where *de novo* tumor initiation and early promotion occur. Analyzing molecular changes in this compartment is most relevant for understanding how VB2 prevents new tumor formation. Future studies profiling treated versus untreated tumors will be valuable to explore VB2’s effects on established tumor cells and complement the current findings.

## 5 Conclusion

To our knowledge, this study firstly confirms *in vivo* that VB2 significantly inhibits hepatic tumorigenesis and establishes a transcriptome database for its anti-tumor mechanism. Mechanistic analysis shows VB2 suppresses liver cancer initiation by enhancing retinol metabolism and inhibiting cell proliferation. Notably, its preventive efficacy against liver tumors markedly exceeds its therapeutic effect, suggesting that VB2 may hold greater value as a chemopreventive agent. These findings provide a novel clinical strategy for preventing postoperative liver cancer recurrence, warranting further research on its application in tumor progression and combination therapies.

## Data Availability

The datasets presented in this study can be found in online repositories. The names of the repository/repositories and accession number(s) can be found in the article/**Supplementary Material**.

## Supplementary material

Supplementary Table 1 Antibodies used for IHC and WB.

Supplementary Table 2 RT-qPCR primer sequences.

Supplementary Table 3 Sample sequencing output data quality evaluation.

Supplementary Table 4 Comparison of clean reads sequence and reference genome.

Supplementary Table 5 Identified DEGs details.

Supplementary Table 6 DEGs enrichment in GO terms details.

Supplementary Table 7 DEGs enrichment in KEGG pathways details.

Supplementary Table 8 GO term enrichment bioinformatics elaboration.

Supplementary Table 9 KEGG pathway enrichment bioinformatic elaboration.

Supplementary Table 10 Exact P-values for the statistical comparisons in the figures.

Supplementary Figure 1 Study workflow. Co., untreated group; VB2, vitamin B2-treated group.

Supplementary Figure 2 VB2 suppresses hepatic tumor progression. **(A)** Illustration of the VB2 dosing regimen in Hras12V mice. **(B)** Representative liver stereogram Images. The red arrow points to the hepatic tumor. **(C)** Comparison of liver weight, body weight, and liver/body weight ratio between control (Co.) and VB2–treated groups. **(D)** Tumors quantification by diameter. **(E)** Serum levels ALT and AST levels in Co. and VB2 groups. M, month; Co., control group; VB2, VB2-treated group; AST, aspartate aminotransferase; ALT, alanine aminotransferase. Data presented as mean ± SEM. **p*<0.05; ***p*<0.01; ****p*<0.001; n=5-8.
