# Supplementary figures and images for: Riboflavin (VB2) inhibits hepatocellular carcinogenesis by enhancing retinol metabolism and suppressing cell proliferation in Hras12V transgenic mice
Source: Front Oncol. 2026 Mar 11;16:1773897. doi: 10.3389/fonc.2026.1773897 (PMC13012928; doi:10.3389/fonc.2026.1773897)

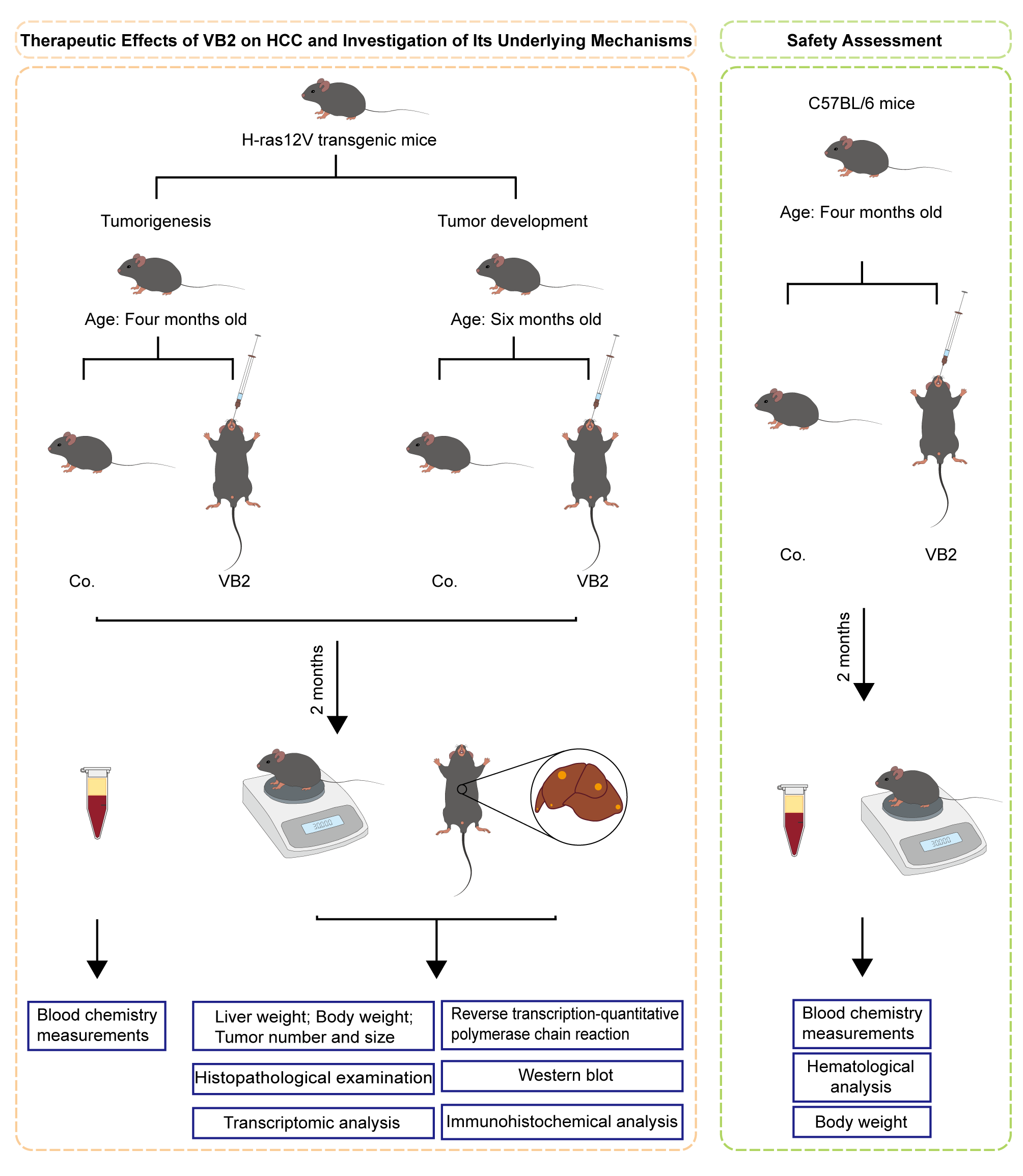

Supplement: Supplementary Figure 1 — Study workflow. Co., untreated group; VB2, vitamin B2-treated group. [file Image1.tif]

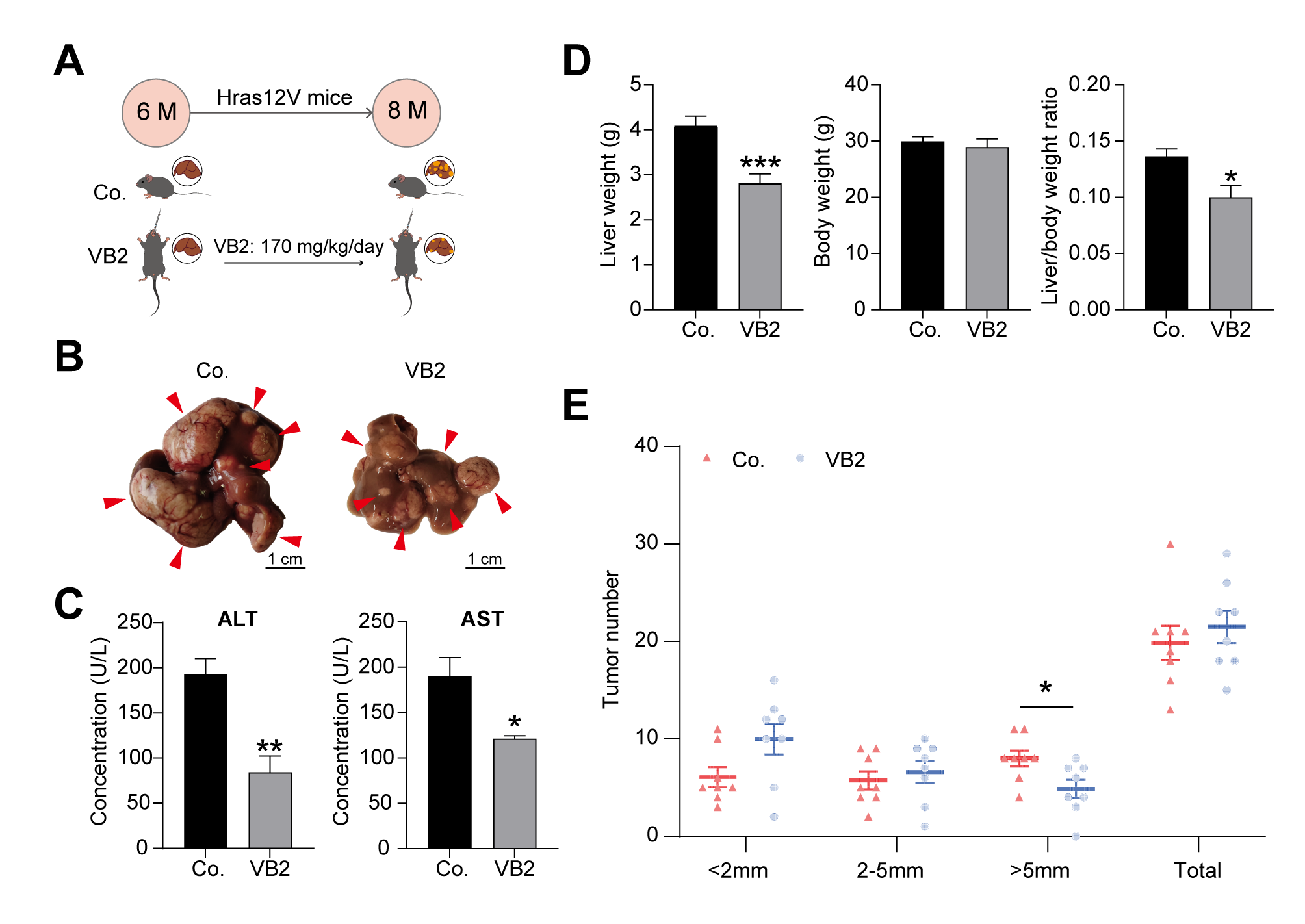

Supplement: Supplementary Figure 2 — VB2 suppresses hepatic tumor progression. (A) Illustration of the VB2 dosing regimen in Hras12V mice. (B) Representative liver stereogram Images. The red arrow points to the hepatic tumor. (C) Comparison of liver weight, body weight, and liver/body weight ratio between control (Co.) and VB2–treated groups. (D) Tumors quantification by diameter. (E) Serum levels ALT and AST levels in Co. and VB2 groups. M, month; Co., control group; VB2, VB2-treated group; AST, aspartate aminotransferase; ALT, alanine aminotransferase. Data presented as mean ± SEM. *p<0.05; **p<0.01; ***p<0.001; n=5-8. [file Image2.tif]
